# Acupuncture for chronic prostatitis/chronic pelvic pain syndrome: study protocol for a randomized controlled trial

**DOI:** 10.1186/s13063-017-2383-8

**Published:** 2017-12-22

**Authors:** Zongshi Qin, Yan Liu, Kehua Zhou, Jiani Wu, Ran Pang, Ning Li, Chang Xu, Joey S. W. Kwong, Zhishun Liu

**Affiliations:** 1 Department of Acupuncture and Neurology, Guang’anmen Hospital, China Academy of Chinese Medical Sciences, Beijing, China; 2 School of Life Sciences, Beijing University of Chinese Medicine, Beijing, China; 3 Data Centre of Traditional Chinese Medicine, China Academy of Chinese Medical Sciences, Beijing, China; 4 Catholic Health System Internal Medicine Training Program, Sisters of Charity Hospital, University at Buffalo, Buffalo, NY USA; 5 Department of Urology, Guang’anmen Hospital, China Academy of Chinese Medical Sciences, Beijing, China; 6 West China Hospital, Sichuan University, Chengdu, China; 7 Chinese Evidence-Based Medicine Center and Chinese Cochrane Center, West China Hospital, Sichuan University, Chengdu, China; 8 JC School of Public Health and Primary Care, Faculty of Medicine, The Chinese University of Hong Kong, Hong Kong, Hong Kong

**Keywords:** Acupuncture, Chronic prostatitis, Chronic pelvic pain syndrome, Randomized controlled trial, Protocol

## Abstract

**Background:**

Chronic prostatitis/chronic pelvic pain syndrome (CP/CPPS) is a common condition affecting men of all ages. Acupuncture may be an effective treatment option for CP/CPPS, but evidence is limited. We propose to evaluate the effectiveness of acupuncture in a rigorously conducted trial.

**Methods:**

Ten hospitals will recruit 440 participants with CP/CPPS in China from October 2017 to December 2019. Participants will be randomly allocated to acupuncture or sham acupuncture with a 1:1 ratio using computerized simple random sampling. The whole study consists of 2-week baseline, 8-week treatment, and 24-week follow up. Twenty 30-mintute sessions of acupuncture or sham acupuncture treatment will be provided between week 1 and 8. The two co-primary outcomes are the proportion of responders at week 8 and week 32. Secondary outcomes include proportion of responders in the two groups at different time points; change in the National Institutes of Health Chronic Prostatitis Symptom Index (NIH-CPSI) total score; change in the NIH-CPSI subscales; change in the International Prostate Symptom Score; change in the Hospital Anxiety and Depression Scale; expectation assessments; proportions of participants in each response category of the Global Response Assessment; change in the International Index of Erectile Function 5; change in the five-level EuroQol five-dimensional questionnaire and a visual analogue scale; and changes in peak and average urinary flow rate.

**Discussion:**

This study will provide robust evidence on whether acupuncture is effective for relieving symptoms of CP/CPPS.

**Trials registration:**

ClinicalTrials.gov, NCT03213938. Registered on 5 July 2017.

**Electronic supplementary material:**

The online version of this article (doi:10.1186/s13063-017-2383-8) contains supplementary material, which is available to authorized users.

## Background

Chronic prostatitis/chronic pelvic pain syndrome (CP/CPPS) refers to the presence of bothersome pelvic pain symptoms without an identifiable cause [1]. Table 1 illustrates the 4 categories of prostatitis per the National Institutes of Health (NIH) classification of prostatitis syndromes. CP/CPPS affects approximately 10–16% of men worldwide [1–4]. Men of all ages can be impacted, among whom those aged 36–50 years are the most commonly affected [5]. There is no apparent racial predisposition to this disease.

**Table 1:** NIH consensus classification and definition of 4 categories of prostatitis [1]

NIH consensus classification of prostatitis	Definition
(I) Acute bacterial prostatitis	Acute infection of the prostate
(II) Chronic bacterial prostatitis	Chronic or recurrent infection of the prostate
(III) Chronic prostatitis/chronic pelvic pain syndrome	No demonstrated infection
IIIA Inflammatory CP/CPPS	Leukocytes in expressed prostatic secretions, post prostate massage urine, or semen
IIIB Non-inflammatory CP/CPPS	No evidence of inflammation
(IV) Asymptomatic inflammatory prostatitis	No subjective symptoms detected, inflammation shown either by prostate biopsy or the presence of leukocytes in EPS/semen during evaluation for infertility or other disorders

*NIH* National Institutes for Health, *CP/CPPS* chronic prostatitis/chronic pelvic pain syndrome

Common symptoms of CP/CPPS include discomfort in the perineum, suprapubic region, and lower urinary tract symptoms [6]. Patients with CP/CPPS also frequently experience a wide array of sexual dysfunctions, including erectile dysfunction (ED), painful ejaculation and premature ejaculation (PE) [7–11], on top of symptoms suggestive of negative cognition or emotional consequences [12]. The aforementioned symptoms negatively impact upon the patient’s quality of life (QoL) to a similar degree or worse than those of congestive heart failure, Crohn’s disease, diabetes mellitus or angina [13, 14]. Because of its high prevalence and lack of effective therapies, direct and indirect costs associated with CP/CPPS are substantial [15]. Approximately 25% of men experience loss of work and approximately 50% have reduced leisure time at some point due to CP/CPPS [16]. The direct and indirect cost of care in China approaches 8059 CNY per person in 2009 [17], and data indicate that in the USA, the total annual cost for patients with prostatitis was US$4387 in 2006 [15].

Unlike acute/chronic bacterial prostatitis, the cause of CP/CPPS is unknown and no well-conducted epidemiologic studies are available to support any particular risk factors [18]. The diagnosis of CP/CPPS is mainly based on symptoms. Treatments for CP/CPPS usually include alpha-blockers, antibiotics, non-steroidal anti-inflammatory drugs (NSAIDs), and phytotherapy. Alpha-blockers and antibiotics have moderate effects on pain, voiding and QoL [19]. Both alpha-blockers and antibiotics are recommended for patients with CP/CPPS for less than 1 year, but only antibiotics are recommended in treatment-naïve patients. NSAIDs and phytotherapy are less commonly used, because of lack of research evidence [19]. Although advances have been made in research and developing novel treatment options, definitive treatments for CP/CPPS are still lacking [19]. Besides, pharmacological interventions are accompanied with adverse effects such as dizziness, nausea, and postural hypotension, which also reduce patients’ compliance to treatments [20]. Previous studies suggest that acupuncture may be a potential treatment for CP/CPPS [21–25]. However, owing to small sample sizes and other methodological limitations of clinical trials, the efficacy of acupuncture in CP/CPPS remains inconclusive [19, 26].

### Aims

The objective of this multi-centre, randomized, sham acupuncture-controlled trial is to assess the effectiveness of acupuncture for relieving symptoms of CP/CPPS.

## Methods

### Study design

We developed this design and protocol according to the guidance for protocols of clinical trials and the standards for reporting clinical trials of acupuncture [27, 28] (see Additional file 1). The framework of this trial consists of two parallel-arm groups, including one acupuncture group and one sham acupuncture group. Details of the intervention procedure will be described in the “Acupuncture group” and “Sham acupuncture group” sections. A data coordination centre will be established at China Academy of Chinese Medical Sciences (CACMS) to monitor data management.

This trial will be performed at 10 hospitals in China. The 10 hospitals are Dongfang Hospital Affiliated to Beijing University of Chinese Medicine, Beijing Fengtai Hospital of Integrated Traditional and Western Medicine, West China Hospital of Sichuan University, the Third Hospital of Zhejiang University of Chinese Medicine, the First Affiliated Hospital of Anhui University of Chinese Medicine, Hengyang Hospital Affiliated to Hunan University of Chinese Medicine, the First Hospital of Hunan University of Chinese Medicine, Guangdong Provincial Hospital of TCM, Yantai Hospital of TCM, and Shanxi Provincial Hospital of TCM. The duration of the study for each participant will be 34 weeks: 2 weeks before randomization as baseline, 8 weeks of treatment, and 24 weeks of follow up.

### Randomization and blinding

The Institute of Basic Research in Clinical Medicine affiliated to CACMS will be responsible for generating the allocation sequence. Participants will be randomly assigned to either the acupuncture or sham acupuncture group using computerized simple random sampling. The allocation ratio is 1:1. Acupuncturists will have access to the information related to participant allocation prior to treatment through a web-response system of Central Randomization System for Clinical Research (designed by the Clinical Evaluation Centre of CACMS). Research assistants, statisticians, and participants will be blind to treatment allocations. The location pf the acupoints and manipulation of needles are similar in the two groups.

**Fig. 1:**
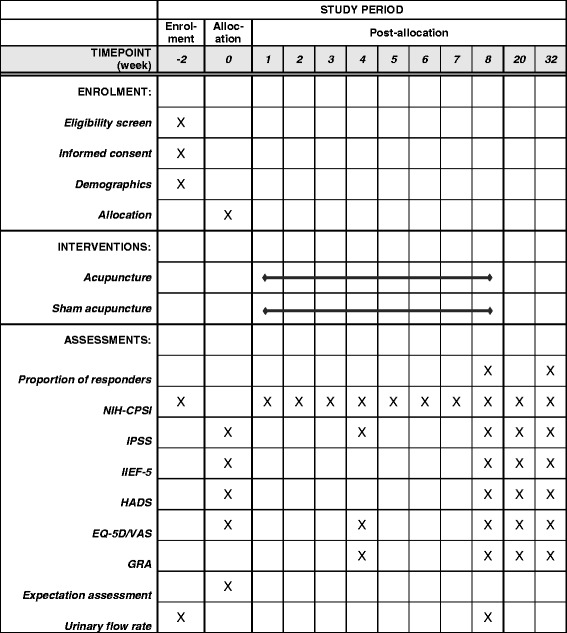
Standard protocol items: recommendation for interventional trials (SPIRIT) schedule of enrolment, interventions, and assessments. *NIH-CPSI* National Institutes of Health Chronic Prostatitis Symptom Index, *IIEF-5* International Index of Erectile Function 5, *HADS* Hospital Anxiety and Depression Scale, *GRA* Global Response Assessment, *EQ-5D-5L* Five-level EuroQol five-dimensional questionnaire, *VAS* visual analogue scale

This trial protocol is developed in accordance with the Declaration of Helsinki [29] and Good Clinical Practice [30], and is registered at www.clinicaltrials.gov (NCT03213938). It has been approved by the Research Ethics Committee of the aforementioned hospitals. Figure 1 illustrates the time schedule of enrolment, interventions, and assessments in this trial.

### Study population and recruitment

We aim to recruit 440 male participants from October 2017 to December 2019. All participants will have to meet the diagnostic criteria for CP/CPPS according to the NIH CP/CPPS consensus: discomfort or pain in the pelvic region for at least 3 months in the previous 6 months. [1]. Urologists will be responsible for the screening, which will include an array of physical examinations (emphasizing rectal examination and ultrasound for the prostate), microbiology of prostatic fluid/urine specimen collected by the “two-glass test” [31], post-void residual urine, urine flow rate, and prostate-specific antigen (PSA).

All participants will be informed by research assistants about the possible benefits and risks associated with this trial, the randomized allocation of treatments, and the enrolment. Participants will be free to withdraw from this trial at any time. Participants will be randomly allocated into the acupuncture or sham acupuncture group. Both traditional Chinese acupuncture and sham acupuncture might be effective for CP/CPPS [21]. Participants will have to sign informed consent before enrolment. The inclusion and exclusion criteria are listed in Table 2 and Table 3, respectively.

**Table 2:** Inclusion criteria

1. History of pain perceived in the prostate region and absence of other lower urinary tract pathology for a minimum of three of the past 6 months. In addition, any associated lower urinary tract symptoms, sexual function, and psychological factors should be addressed. Physical examinations, urine analyses, and urine cultures will be performed for all subjects
2. Age 18 to 50 years.
3. NIH Chronic Prostatitis Symptom Index (NIH-CPSI) total score ≥15

**Table 3:** Exclusion criteria

1. Prostate, bladder, or urethral cancer, seizure disorder in any medical history
2. Inflammatory bowel disease, active urethral stricture, neurologic disease or disorder affecting the bladder, liver disease, neurologic impairment or psychiatric disorder preventing understanding of consent and self-report scale
3. Urinary tract infection with a urine culture value >100,000 colony forming units (CFU)/mL, clinical evidence of urethritis, including urethral discharge or positive culture, diagnostic of sexually transmitted diseases (including gonorrhoea, chlamydia, mycoplasma or trichomonas, but not including HIV/AIDS), symptoms of acute or chronic epididymitis
4. Residual urine volume ≥100 mL
5. Qmax ≤15 mL/s
6. During previous 4 weeks used androgen hormone inhibitors (finasteride), alpha-blockers (terazosin, doxazosin mesylate, tamsulosin hydrochloride), antibiotics (ciprofloxacin hydrochloride), or any other prostatitis-specific medication (including herbal and Chinese medicine)

### Acupuncture group

The treatment protocol was developed by consensus meeting with acupuncturists and acupuncture experts from CACMS. Sanyinjiao (SP6), Zhongliao (BL33), Shenshu (BL23), and Huiyang (BL35) were selected for the acupuncture treatment group. Based on the theories of traditional Chinese medicine (TCM) and acupuncture, the spleen meridian (SP), kidney meridian (KI), and liver meridian (LI) intersect at Sanyinjiao (SP6), one of the most frequently used acupoints for urological disorders. The urinary bladder meridian (BL) is commonly used for urologic disorders, including pelvic pain, lower urinary tract symptoms, and sexual disorders in both male and female patients [32]. SP6 locates slightly above the medial malleolus, posterior to the tibia where the tibial nerve innervates anatomical structures. Previous research supports the possible beneficial effects of sacral nerve stimulation and tibial nerve stimulation on the severity and frequency of chronic pelvic pain [33, 34].

With patients in a prone position in relaxation, acupuncturists will use 75% alcohol pads to sterilize the skin around the acupuncture pointsand then insert Hwato-brand disposable acupuncture needles (sizes 0.30 × 75 and 0.30 × 40 mm) into the acupuncture points. For bilateral Zhongliao (BL33), the needle will be inserted approximately 50–60 mm with a 30° to 45° angle in an inferomedial direction. For Huiyang (BL35), the needle will be inserted approximately 50–60 mm in a slightly superolateral direction. For Shenshu (BL23) and Sanyinjiao (SP6), the needles will be inserted vertically with a depth of 25–30 mm. For acupuncture at BL23, BL35 and SP6, needle insertion will be followed by acupuncture manipulation of lifting and thrusting combined with twirling and rotating to reach *deqi*, which is defined as the sensation of aches, soreness, swelling, heaviness or numbness in the needle location region [35]. Manipulations will be performed once every 10 minutes, 30 seconds each time. Immediately after needle removal, the acupuncturist will gently press the needled skin area with dry sterilized cotton balls to avoid bleeding.

### Sham acupuncture group

The participants in the sham acupuncture group will receive shallow needling at bilateral sham BL23, BL33, BL35 and SP6. Treatment protocol will be similar to that of the acupuncture group, but Hwato-brand disposable acupuncture needles (size 0.30 × 25 mm) will be inserted at non-acupuncture points lateral to the corresponding acupoints (15 mm to BL23, BL33 and BL35; 10 mm to SP6) with depths of 2–3 mm without manipulation [36].

Both treatment groups will receive 20 sessions of treatment over an 8-week period after baseline (3 sessions in each of the first 4 weeks, and 2 sessions in each of the remaining 4 weeks). Each session will last for 30 minutes. Guang’anmen Hospital will supply the acupuncture needles. In each center, two acupuncturists will provide acupuncture or sham acupuncture treatment. To improve the consistency of treatments, acupuncturists will receive trial-specific (standardized operation procedure) training prior to performing treatments. The training includes a video showing detailed information on how to perform the acupuncture and sham acupuncture.

### Blinding assessment

To test the success of blinding, participants will be asked to reply to the following question at the 8th week of treatment (sessions 19 or 20): “Do you think you have received traditional acupuncture in the past weeks?” The participants will be able to choose one of the following options as the answer: “Yes”, “No” or “Unclear”.

### Rescue medicine

The use of medications or other therapies for symptoms of CP/CPPS will be discouraged during this trial. However, medication use is allowed for intolerable symptoms as long as it is recorded accordingly, including the name, the dosage, and the duration of the medication use. We will compare the proportion of subjects using rescue medications and the duration of medication use between groups.

### Outcome measurement

The two co-primary outcomes include the proportions of responders at week 8 and week 32. The responder is defined as a participant with a decline of 6 points or more from baseline in the total score of the Chinese version National Institutes of Health Chronic Prostatitis Symptom Index (NIH-CPSI). As a validated, self-reported questionnaire, NIH-CPSI is widely used to assess CP/CPPS symptoms [37, 38]. NIH-CPSI consists of 9 items divided into 3 discrete domains: pain (0–21 points), urinary symptoms (0–10 points) and QoL (0–12 points), with a total score of 0–43 points (a higher score indicates worse symptoms). A decline of at least 6 points in NIH-CPSI has been identified as the minimal clinically important difference (MCID) [25].

Secondary outcomes include (a) the proportion of responders in the two groups at other time points; (b) change in the NIH-CPSI total score; (c) change in the NIH-CPSI subscales, including pain, urinary symptoms, and QoL impact; (d) change in the International Prostate Symptom Score (IPSS); (e) change in the Hospital Anxiety and Depression Scale (HADS); (f) expectation assessments; (g) the proportions of participants in each response category of the Global Response Assessment (GRA); (h) changes in peak and average urinary flow rate; (i) change in the score of the International Index of Erectile Function 5 (IIEF-5); (j) change in the five-level EuroQol five-dimensional questionnaire (EQ-5D-5L) and a visual analogue scale (VAS). The NIH-CPSI-related secondary outcomes (items a–c) will be assessed once a week during treatment, and at the 12th to 24th weeks after treatment. IPSS will be assessed at the end of the 4th, 8th, 20th, and 32nd week. IPSS is a valid, reliable and sensitive measure for patients with lower urinary tract symptoms (LUTS); it is widely used in clinical practice and research to determine the severity of LUTS, including incomplete bladder emptying, frequency of urination, intermittency, urgency, weak urine stream, straining and nocturia [39, 40]. Each of the questions is rated from 0 (not at all) to 5 (almost always); severity of LUTS can be graded as mild (0–7), moderate (8–19) or severe (20–35). HADS will be assessed at the end of the 8th, 20th, and 32nd week. HADS is made up of 7 items for the assessment of depression and anxiety; the completion of this scale usually requires 2–5 minutes [41].

Expectation assessment will be assessed at baseline; it includes two brief questions to investigate whether patients are confident that acupuncture treatment will help their CP/CPPS: “In general, do you believe acupuncture is effective for treating the illness?”, “Do you think acupuncture will be helpful to improve your CP/CPPS symptoms?” For each question, participants will choose “Yes”, “No”, or “unclear” as the answer. The proportions of participants in each response category of the GRA in the two groups after treatment will be measured at the end of the 4th, 8th, 20th, and 32nd week. GRA consists of 7 response categories: markedly worsened, moderately worsened, slightly worsened, no change, slightly improved, moderately improved, and markedly improved. We will identify a participant who reports “moderate” or “marked improvement” as a responder [42]. Peak and average urinary flow rates will be measured at the end of treatment (8th week). The International Index of Erectile Function 5 (IIEF-5) Chinese version will be assessed at the end of the 8th, 20th, and 32nd week. The IIEF-5 consists of 15 items in 5 domains with a total score ranging from 5 to 25 (mild ED, 12–21; moderate ED, 8–11; severe ED, 5–7); it is a psychometrically valid and reliable instrument with high sensitivity and specificity for detecting treatment effects in patients with ED of a broad spectrum of aetiology [43]. The EQ-5D-5L and VAS will be assessed at the end of the 4th, 8th, 20th, and 32nd week. The EQ-5D is a generic instrument designed for self-completion and postal surveys; it is well-established and suitable for evaluation of QoL in participants with CP/CPPS [44].

We will provide a self-assessment questionnaires/scales notebook for each participant. Participants will be asked to complete the notebook at home weekly or monthly, and bring the manual to hospital the following week or month. After collecting the manual, data from the notebook will be entered into the electronic database, in which, the data from the notebook will be the raw data for analysis.

### Safety assessment

We will handle and document the adverse events (AEs) using the standard operating procedures for monitoring and reporting all AEs. According to their potential association with the treatment, AEs will be categorized as treatment-related or non-treatment-related within 24 hours after their occurrence. Treatment-related AEs include pain, haematoma, localized infection, broken needle, fainting, nausea, headache, dizziness, insomnia, vomiting, or palpitations during or after treatment. Any serious adverse events (SAEs) will be immediately reported to the principal investigator (ZL) and the Medical Ethics Committee within 24 hours. A research assistant will be required to record SAEs, including information on the time of occurrence, severity, duration, measurement, management, and its outcome. Guang’anmen Hospital has insurance cover for harm associated with the interventions during this trial.

### Quality control

All practitioners, including acupuncturists, research assistants, and statisticians, will be required to attend training to ensure the quality of this trial. All acupuncturists in this trial have completed their professional training in acupuncture in universities of Chinese medicine with more than 2 years clinical experience. The interventions will be performed based on rigorous adherence to the standardized operating procedure.

### Calculation of sample size and statistical analyses

The primary study hypothesis is that acupuncture is more effective than sham acupuncture in relieving symptoms of CP/CPPS. Sample-size calculations were based on 90% power to detect a difference of 17% between response rates in the two groups (63.7% in the acupuncture group compared with 46.7% in the control group) for the primary outcome, defined as a decline of 6 or more points in the NIH-CPSI total score [21]. These values are equivalent to an odds ratio of 2.0. On the basis of a two-sided alpha level of 0.05, we calculated that a sample of 440 participants will be required (220 in each group). This proposed sample size includes a 15% increase to account for dropouts. To control for type I error, the two time points will have to be positive in order for the trial to prove the efficacy of acupuncture.

Summary tables (descriptive statistics and/or frequency tables) will be provided for all variables as appropriate. Means and standard deviations will be presented for continuous variables, unless the variable has a skewed distribution, in which case medians, and 25th and 75th percentiles will be presented. The number and percentage of participants in each category will be presented for categorical variables. Missing values will be reported for each variable (continuous or categorical). All randomized participants will be included in the analyses. All analyses will be based on the intention-to-treat principle.

Primary outcome analysis will be conducted with a logistic generalized linear mixed model (GLMM) for repeated measures. Response or non-response at each scheduled post-baseline visit (end of treatment, and 20 and 24 weeks after treatment) is the dependent variable. Subjects who discontinue without providing a post-baseline NIH-CPSI score will be considered non-responders at visit 1 (week 1). The logistic GLMM is fit using the logit link and the binomial distribution. The model will include the baseline NIH-CPSI total score as a fixed-effect covariate, with fixed-effect categorical factors for a treatment group (acupuncture and sham acupuncture), visit and treatment × visit interaction. The interaction will remain in the model regardless of significance. Treatment group comparisons at each visit will be estimated by differences between least squares means from the treatment × visit interaction, and will be presented as odds ratios with accompanying *p* values and 95% CIs. The predicted probability of response at each visit will also be presented. Such models provide fairly robust results for treatment comparisons when longitudinal binary responses are missing, with an assumption that data are missing at random, according to reported values [45]. Sensitivity analysis will be performed if necessary, with a control-based pattern imputation model under the assumption of missing not at random.

Changes from baseline in the NIH-CPSI total score will be analyzed using linear mixed-effects models. The observed change from baseline score at each assessment point will be considered as the dependent variable. The model will include the baseline value, treatment group (acupuncture and sham acupuncture), visit and treatment × visit interaction. Treatment group comparisons at each visit will be estimated by differences between least squares means from the treatment × visit interaction, with accompanying *p* values and 95% CIs. Log-transformation may be applied in the case of serious violations of the model assumptions (normality and constant variance of the residuals). If not appropriate, the Wilcoxon rank-sum test will be used. The effect of the treatment will be estimated by the difference (or ratio, in the case of log-transformation) between treatments and will be presented along with its associated 95% CI. The same approach as for the NIH-CPSI total score will be used in other longitudinal continuous outcomes such as NIH-CPSI subscales (pain, urinary symptoms, and QoL).

Other categorical data or ordinal data will be compared between groups using the Wilcoxon rank-sum test, chi-square test or Fisher’s exact test, as appropriate. The James and Bang blinding indices will be used to assess the success of blinding. The James blinding index is a variation on the statistic in which 1 represents perfect blinding. The Bang blinding index for each group represents the proportion of participants making a correct treatment guess beyond chance; 0 represents perfect blinding, a positive index indicates a correct guess, and a negative index indicates a guess in the opposite direction [46]. For all statistical analyses, SAS 9.4 software will be used. All hypothesis testing will be carried out at the 5% (two-sided) significance level.

## Discussion

The nomenclature of CP/CPPS indicates that this disease is a poorly understood pain syndrome that may be unrelated to the prostate, at least in some men. To date, research efforts have failed to provide any consistent link between symptoms and any prostate pathology. Thus, this term is also used to describe men with pelvic symptoms of unclear aetiology. According to the NIH CP/CPPS classification, it could be divided into two subgroups, CP/CPPS IIIA and CP/CPPS IIIB, representing patients with an unspecified level of leukocytes in prostate secretions and patients without leukocytes in prostate secretions, respectively. In this trial, we will not divide patients into types IIIA and IIIB because assessment of leukocyte is of unclear significance to patients.

The NIH-CPSI was developed specifically for the evaluation of men in clinical trials. A 6-point decrease in NIH-CPSI total score could serve as the optimal threshold to predict response [25]. In previous well-designed RCTs comparing drugs to a placebo, a 4-point decrease in the NIH-CPSI was set as a threshold to predict the response rate [42, 47]. This study also uses this valid and reliable scale in primary outcome assessments to compare the response rate between acupuncture and sham acupuncture groups. We set a 6-point instead of a 4-point decrease as the threshold to identify responders. This may help substantiate the efficacy of acupuncture for CP/CPPS, if any, as the optimal threshold to predict response remains to be established. Based on available data, a 4-point decline has sensitivity of 90% and specificity of 60%, and a 6-point decline has sensitivity of 77% and specificity of 71%; however, receiver operating characteristic (ROC) curves suggest that a cut point of 6 points has better discriminative ability in distinguishing responders from non-responders [25].

Several RCTs investigating acupuncture versus sham acupuncture or drugs have been published [21, 22]. Although the findings of these acupuncture studies support the therapeutic effects of acupuncture/electro-acupuncture for relieving symptoms of CP/CPPS, small sample sizes, different types of interventions, and statistical limitations limit the quality of evidence. Besides, owing to the different types of acupuncture used or different control groups in these studies, these previous RCTs could not be synthesised appropriately in meta-analyses. As a result, the efficacy of acupuncture for CP/CPPS remains inconclusive. In this trial, we will use sham acupuncture to evaluate the efficacy of acupuncture for relieving symptoms of CP/CPPS. With the standard outcome measurements in chronic prostatitis, the larger sample size, the 24-week follow-up period, and the strict quality control, our study will provide robust evidence on whether there are clinical benefits of acupuncture to patients with CP/CPPS. Nonetheless, this trial will have some limitations: the acupuncturist will not be blinded to treatment allocation, the location of sham acupoints are close to real acupoints, and the sham acupuncture needle will penetrate subcutaneously.

## Trial status

No recruitment at the present.

## Additional file

Additional file 1: SPIRIT checklist. (DOC 257 kb)

## Abbreviations

AEs: Adverse events
BL: Bladder meridian
CACMS: China Academy of Chinese Medical Sciences
CIs: Confidence intervals
CNY: China Yuan
CP/CPPS: Chronic prostatitis/chronic pelvic pain syndrome
EAU: European Association of Urology
ED: Erectile dysfunction
EPS: Expressed prostatic secretion
EQ-5D-5L: Five-level EuroQol five-dimensional questionnaire
GLMM: Generalized linear mixed model
GRA: Global Response Assessment
HADS: Hospital Anxiety and Depression Scale
IIEF-5: International Index of Erectile Function 5
IPSS: International Prostate Symptom Score
KI: Kidney meridian
LI: Liver meridian
LUTS: Lower urinary tract symptoms
NIH: National Institutes of Health
NIH-CPSI: National Institutes of Health Chronic Prostatitis Symptom Index
NSAIDs: Non-steroidal anti-inflammatory drugs
PSA: Prostate-specific antigen
QoL: Quality of life
RCT: Randomized controlled trial
SAEs: Serious adverse events
SP: Spleen meridian
TCM: Traditional Chinese medicine
VAS: Visual analogue scale
